# CtsR, the Master Regulator of Stress-Response in *Oenococcus oeni*, Is a Heat Sensor Interacting With ClpL1

**DOI:** 10.3389/fmicb.2018.03135

**Published:** 2018-12-18

**Authors:** Maud Darsonval, Frédérique Julliat, Tarek Msadek, Hervé Alexandre, Cosette Grandvalet

**Affiliations:** ^1^UMR A. 02.102 Procédés Alimentaires et Microbiologique, AgroSup Dijon, Université Bourgogne Franche-Comté, Dijon, France; ^2^Unité de Biologie des Bactéries Pathogènes à Gram Positif, Institut Pasteur, Paris, France; ^3^CNRS ERL 6002, Paris, France; ^4^Institut Universitaire de la Vigne et du Vin – Jules Guyot, Dijon, France; ^5^Institut National Supérieur des Sciences Agronomiques, de L′Alimentation et de L′Environnement, AgroSup Dijon, Dijon, France

**Keywords:** *Oenococcus oeni*, stress response, CtsR, RNA silencing, heterologous expression system, two-hybrid system

## Abstract

*Oenococcus oeni* is a lactic acid bacterium responsible for malolactic fermentation of wine. While many stress response mechanisms implemented by *O. oeni* during wine adaptation have been described, little is known about their regulation. CtsR is the only regulator of stress response genes identified to date in *O. oeni.* Extensively characterized in *Bacillus subtilis*, the CtsR repressor is active as a dimer at 37°C and degraded at higher temperatures by a proteolytic mechanism involving two adapter proteins, McsA and McsB, together with the ClpCP complex. The *O. oeni* genome does not encode orthologs of these adapter proteins and the regulation of CtsR activity remains unknown. In this study, we investigate CtsR function in *O. oeni* by using antisense RNA silencing *in vivo* to modulate *ctsR* gene expression. Inhibition of *ctsR* gene expression by asRNA leads to a significant loss in cultivability after heat shock (58%) and acid shock (59%) highlighting the key role of CtsR in the *O. oeni* stress response. Regulation of CtsR activity was studied using a heterologous expression system to demonstrate that *O. oeni* CtsR controls expression and stress induction of the *O. oeni hsp18* gene when produced in a *ctsR*-deficient *B. subtilis* strain. Under heat stress conditions, *O. oeni* CtsR acts as a temperature sensor and is inactivated at growth temperatures above 33°C. Finally, using an *E. coli* bacterial two-hybrid system, we showed that CtsR and ClpL1 interact, suggesting a key role for ClpL1 in controlling CtsR activity in *O. oeni*.

## Introduction

*Oenococcus oeni* is an acidophilic wine-associated lactic acid bacterium (LAB), mainly responsible for malolactic fermentation (MLF) of wine, usually following yeast-driven alcoholic fermentation (Lonvaud-Funel, 1999). Wine and the winemaking process form a harsh and challenging environment combining stresses such as low pH (3–3.5), low temperatures (14–18°C), the presence of ethanol, nutrient starvation and competing organisms (yeasts) generating abiotic growth inhibitors (ethanol, sulfites, decanoic, and dodecanoic acids). Like most microorganisms facing stress conditions, *O. oeni* must adapt to survive and deciphering the molecular mechanisms involved in responding to stress is an important step to improve *O. oeni* MLF performance and design future malolactic starter strains. Because of its acidophilic profile and its unique genome organization, *O. oeni* is an intriguing and challenging model to investigate stress response mechanisms in LAB (Bartowsky, 2017; Grandvalet, 2017). Over the past decades, several genetic responses adopted by *O. oeni* during wine adaptation have been described, including genes involved in general stress response, membrane composition and fluidity, pH homeostasis, oxidative stress response, presence of sulfites and DNA damage (Salema et al., 1996; Guzzo et al., 1997, 1998; Jobin et al., 1997, 1999; Tourdot-Maréchal et al., 2000; Da Silveira et al., 2003; Beltramo et al., 2004; Chu-Ky et al., 2005; Coucheney et al., 2005b; Grandvalet et al., 2005, 2008; Da Silveira and Abee, 2009; Maitre et al., 2014; Darsonval et al., 2016; Margalef-Català et al., 2016).

*Oenococcus oeni* stress response mechanisms involve the synthesis of Heat Shock Proteins (HSPs), a universal stress response with several regulatory pathways described in *Firmicutes*. Indeed, *hsp* genes can be induced by the alternative sigma factor σ^B^ or repressed by transcriptional repressors such as CtsR or HrcA (Benson and Haldenwang, 1993; Schulz and Schumann, 1996; Derré et al., 1999; Schumann, 2003). In Streptococci, HrcA and CtsR control two partially overlapping regulons that include most *hsp* genes (Chastanet et al., 2001; Chastanet and Msadek, 2003; Grandvalet et al., 2005; Spano and Massa, 2006; Frees et al., 2007). In contrast, in *Bacilli*, the two regulons are entirely distinct while in *Staphylococci* the HrcA regulon is completely embedded within the CtsR regulon (Chastanet et al., 2003). Both transcriptional repressors control expression of their regulon by specifically binding to their operator sequences in the promoter region, preventing RNA polymerase recruitment. HrcA specifically recognizes the CIRCE (“Controlling inverted repeat of chaperone expression”) palindromic sequence while CtsR binds a tandemly repeated hepta-nucleotidic operator sequence (Zuber and Schumann, 1994; Derré et al., 1999). CtsR is the only regulator of stress response gene expression identified and studied in *O. oeni* so far (Grandvalet et al., 2005). The sequence analysis of the scaffold *O. oeni* ATCC BAA-1163 genome^1^ allowed the identification of the *ctsR* gene sequence and CtsR operator sequences, but no *hrcA* gene, CIRCE sequences or other genes encoding known stress response regulators have been found. Likewise in *O. oeni* PSU-1 (NC_008528), the only complete sequenced strain, no gene encoding known regulators of stress response have been identified, except *cstR*. In contrast, six two-component systems (TCS), some of which may be involved in stress response, have been identified in the *O. oeni* genome (Zúñiga et al., 2011). In *Firmicutes*, most molecular chaperone genes (*dnaK, groEL*) and *clp* genes, encoding either ClpATPases and ClpP protease, have CtsR operator sites in their promoter region. To date, *O. oeni* is the only *Firmicutes* where expression of *dnaK* and *groEL* is controlled exclusively by CtsR and not by HrcA (Grandvalet et al., 2005). In *Bacillus subtilis*, CtsR is active as a dimer under optimal growth conditions and represses transcription of its regulon by binding its operator sequence (Derré et al., 1999, 2000). Under stress conditions, the CtsR dimer is phosphorylated by McsA and McsB and then recognized and degraded by the ClpCP proteolytic complex (Derré et al., 2000; Kirstein et al., 2005, 2007; Elsholz et al., 2010, 2011). LAB are *mcsAB*-deficient *Firmicutes* and alternative mechanisms for regulating the CtsR activity have been described. In *Lactococcus lactis*, ClpE is required to restore repression by CtsR after heat shock. Indeed, replacement of *clpE* by *mcsA* was shown to restore *hsp* gene repression suggesting that ClpE in *L. lactis* has the same function as McsA in *B. subtilis* by interacting with CtsR through its zinc finger motif (Varmanen et al., 2003). More recently, Tao and Biswas (2013) showed that the ClpCP complex is not required for specific degradation of CtsR in *Streptococcus mutans* but that ClpL displays a chaperone protective role helping CtsR to bind its operator sequence (Tao et al., 2012; Tao and Biswas, 2013). In addition, in *L. lactis, Geobacillus stearothermophilus*, and *B. subtilis*, CtsR has been shown to act directly as a heat sensor with distinct species-specific thermal derepression temperature thresholds (Elsholz et al., 2010). The *O. oeni* genome does not contain *clpE*, *mcsA*, and *mcsB* genes, however two *clpL* genes are present: *clpL1*, in an operon with *clpP*, and *clpL2* (Beltramo et al., 2006; Assad-García et al., 2008). This strongly suggests a likely involvement of ClpL1 and/or ClpL2 in the regulation of CtsR activity in *O. oeni*. *O. oeni* is not readily genetically tractable, few genetic tools are available and none for directed mutagenesis or gene deletion. Because of these technical barriers, the *in vivo* function of CtsR and the regulatory mechanisms controlling its activity in *O. oeni* remain unknown.

In this study, we first investigated the *in vivo* role of CtsR in *O. oeni* using antisense RNA silencing, a technique we recently used to show the first modulation of gene expression in *O. oeni* and confirm the molecular chaperone role of the small Hsp Lo18 (Darsonval et al., 2016). Using this approach, we inhibited *ctsR* gene expression by producing a full-length antisense RNA (asRNA) of the *ctsR* mRNA. We then used *B. subtilis* 168 as a tool to explore regulation of CtsR activity. We combined a xylose-inducible heterologous expression system and a β-galactosidase reporter system based on a transcriptional fusion with the *O. oeni hsp18* promoter to measure repression by *O. oeni* CtsR at different temperatures. Finally, we tested protein–protein interactions between CtsR and target Clp ATPases using an *E. coli* two-hybrid system (Karimova et al., 1998) to identify direct partners of CtsR.

## Materials and Methods

### Bacterial Strains and Growth Conditions

Bacterial strains used in this study are listed in Table 1.

**Table 1:** Bacterial strains or plasmids used in this study.

Strain or Plasmid	Relevant genotype or description	Source or reference

**Strains**		
***Escherichia coli***		

EC101	*E. coli* JM101(*supEthi (lacproAB) (*F’ *traD36 proABlacI^q^ Z*Δ*M15*) with *repA* from pWV01 integrated in chromosome	Laboratory stock (Law et al., 1995)
C2992I	*E. coli* DH5α F̀ *proA^+^B^+^ lacIq* Δ*(lacZ)M15 zzf::Tn10/ fhuA2*Δ*(argF-lacZ)U169 phoA glnV44 Φ80*Δ*(lacZ)M15 gyrA96 recA1 relA1 endA1 thi-1 hsdR17*, tet^r^	New England Biolabs Inc.,
EcASCtsR	*E. coli* EC101 carrying pSIPSYNCtsR	This study
Ecsyn	*E. coli* EC101 carrying pSIPSYN	(Darsonval et al., 2016)
BL21	*E. coli* F^−^ *dcm ompT* Δ*hsdS* (r_B_- m_B_-) *gal* [*malB*^+^]_K-12_(λ^S^)	Thermo Fisher Scientific
BL21-CtsR	BL21 carrying pET*ctsR*25	This study
BL21-Lo18	BL21 carrying pET*hsp18*	This study
DHT1	F^−^*glnV44*(AS) *recA1 endA1 gyrA96* (*nal^r^*) *thi-1 hsdR17 spoT1 rfbD1 cya^−^854ilv-691*::Tn*10*	(Dautin et al., 2000)
DHT1 zip-zip	*E. coli* DHT1 harboring pT25-zip and pT18-zip encoding the GCN4 leucine zipper	(Karimova et al., 2000)
***Oenococcus oeni***		

*O. oeni* ATCC BAA-1163	wild type strain, van^r^	Laboratory stock
OoASctsR	*O. oeni* ATCC BAA-1163, pSIPSYNASCtsR	This study
Oosyn	*O. oeni* ATCC BAA-1163, pSIPSYN	(Darsonval et al., 2016)
***Bacillus subtilis***		

168	Wild type*, trpC2*	Laboratory stock
QB4991	*trpC2 amyE::(‘lacZ aphA3)* Δ*ctsR*	(Derré et al., 1999)
WT	*trpC2 amyE ::*(*hps18’-bgaB cat)*	pDL*hsp18* →168 (Grandvalet et al., 2005)
Δ*ctsR*	*trpC2* Δ*ctsR amyE ::*(*hps18’-bgaB cat)*	pDL*hsp18* → QB4991 (Derré et al., 2000; Grandvalet et al., 2005)
Δ*ctsR-*XT*OoctsR* pXTOoCtsR	*trpC2*, Δ*ctsR amyE::*(*hps18’-bgaB cat), thrC::(*p*xylA-ctsR-O. oeni_-_spc*)	pXTctsR25 → Δ*ctsR-hsp18’*

**Plasmids**		

pSIPSYN	Replicative and low-copy number plasmid allowing gene expression in *O. oeni* under the control of P_SY N_ promoter, ery^R^	(Darsonval et al., 2016)
pSIPSYNASCtsR	pSIPSYN derivative encoding the *ctsR* ORF in antisense orientation under the control of P_SY N_ promoter	This study
pET28a(+)	Vector for expression of N-terminal His-tagged proteins in BL21 under the control of pT7 promoter, kan^R^	Novagen
pET*ctsR*	pET28a(+) derivative carrying the *O. oeni ctsR* coding sequence, kan^R^	This study
pET*hsp18*	pET28a(+) derivative carrying the *O. oeni hsp18* coding sequence.	This sudy
pXT	pDG1728 derivative allowing transcriptional fusion with P_xylA_ xylose inducible promoter and integration into the *B. subtilis thrC* locus, spec^R^, amp^R^	(Derré et al., 2000)
pXT*ctsR*25	pXT derivative carrying the *O. oeni ctsR* coding sequence under the control of P_xylA_ xylose inducible promoter	This study
pDL	Integrative plasmid for constructing transcriptional fusions with the *G. stearothermophilus bgaB* gene and integration into the *B. subtilis amyE* locus, cm^R^	(Yuan and Wong, 1995)
pDL*hsp18*	pDL derivative with transcriptional fusion *hsp18’*-*bgaB*	(Grandvalet et al., 2005)
pKT25	BACTH vector designed to express in DHT1 a given polypeptide fused in frame at its N-terminal end with T25 fragment, kan^R^	(Karimova et al., 2000)
pKNT25	BACTH vector designed to express in DHT1 a given polypeptide fused in frame at its C-terminal end with T25 fragment, kan^R^	(Karimova et al., 2000, 2005)
pUT18C	BACTH vector designed to express in DHT1 a given polypeptide fused in frame at its N-terminal end with T18 fragment, amp^R^	(Karimova et al., 2000)
pUT18	BACTH vector designed to express in DHT1 a given polypeptide fused in frame at its C-terminal end with T18 fragment, amp^R^	(Karimova et al., 2000)
pKT25-*ctsR*	Full-length *ctsR*ORF cloned into pKT25	This study
pKT25-*clpC*	Full-length *clpC*ORF cloned into pKT25	This study
pKT25-*clpL1*	Full-length *clpL1*ORF cloned into pKT25	This study
pKT25-*clpL2*	Full-length *clpL2* ORF cloned into pKT25	This study
pKNT25-*ctsR*	Full-length *ctsR* ORF without stop codon cloned into pKNT25	This study
pKNT25-*clpC*	Full-length *clpC* ORF without stop codon cloned into pKNT25	This study
pKNT25-*clpL1*	Full-length *clpL1* ORF without stop codon cloned into pKNT25	This study
pKNT25-*clpL2*	Full-length *clpL2* ORF without stop codon cloned into pKNT25	This study
pUT18C-*ctsR*	Full-length *ctsR* ORF cloned into pUT18C	This study
pUT18C-*clpC*	Full-length *clpC* ORF cloned into pUT18C	This study
pUT18C-*clpL1*	Full-length *clpL1* ORF cloned into pUT18C	This study
pUT18C-*clpL2*	Full-length *clpL2* ORF cloned into pUT18C	This study
pUT18-*ctsR*	Full-length ctsR ORF without stop codon cloned into pUT18	This study
pUT18-*clpC*	Full-length *clpC* ORF without stop codon cloned into pUT18	This study
pUT18-*clpL1*	Full-length *clpL1* ORF without stop codon cloned into pUT18	This study
pUT18-*clpL2*	Full-length *clpL2* ORF without stop codon cloned into pUT18	This study

*van^r^, vancomycin resistance; ery^r^, erythromycin resistance; cm^r^, chloramphenicol resistance; spec^r^, spectinomycin resistance; amp^r^, ampicillin resistance; kan^r^, kanamycin resistance; tet^R^, tetracyclin resistance.*

*Oenococcus oeni* ATCC BAA-1163 is an acidophilic strain isolated in Aquitaine (France) from red wine (Lonvaud-Funel, 1999). *O. oeni* was grown at 28°C in FT80m medium (pH 5.3) (Cavin et al., 1989) supplemented with 20 μg ⋅ ml^−1^ of vancomycin, 20 μg ⋅ ml^−1^ of lincomycin and 20 μg ⋅ ml^−1^ of erythromycin when required. For stress survival tests, cells were harvested during late exponential phase (OD_600_ = 0.8 corresponding to 1.10^8^ CFU ⋅ ml^−1^) and directly transferred to 48°C for heat stress or into acidified FT80m medium (pH 3.5) then incubated at 28°C during 90 min. Bacterial cultivability was estimated on FT80m agar plates (CFU ⋅ ml^−1^) supplemented with relevant antibiotics. Growth was monitored in liquid culture by following OD_600_ over time.

*Escherichia coli* EC101 and C2992I were used as host strains for cloning and plasmid maintenance. Bacterial adenylate cyclase two-hybrid (BACTH) assays were carried out with the *E. coli cya^−^* strain DHT1, kindly provided by MPSDM. *E. coli* strains were grown at 37°C (except DHT1, 28°C) in Luria-Bertani (LB) medium supplemented with erythromycin (250 μg ⋅ l^−1^), kanamycin (50 μg ⋅ ml^−1^), or/and ampicillin (100 μg ⋅ ml^−1^) when necessary.

*Bacillus subtilis* 168, *ctsR-*deficient QB4991 and derivative strains were grown at 37°C in LB medium supplemented with chloramphenicol (5 μg ⋅ ml^−1^) and spectinomycin (100 μg ⋅ ml^−1^) when required.

**Table 2:** Primers used in this study.

Primer^1^	Sequence (5′–3′)^2,3^	Plasmid construction and function	Restriction site	Source or reference
**RNA silencing in *O. oeni***				

ASctsR3	CGTCCCGGGATGGCAGAAGCTAATATTTCAGAT	pSIPSYNASCtsR: *ctsR* coding sequence for antisense cloning in pSIPSYN	*Sma*I	This study
ASctsR6	GGGCCATGGCAGATGCTGTGTATTGATTATCCA		*Nco*I	This study

**Heterologous expression and reporter system in *B. subtilis***				

ctsR2	CGGACTAAGCTTTTATCCATGAATGTTTGTACTCT	pXTCtsR25: *O. oeni ctsR* coding sequence into pXT for heterologous expression in *B. subtilis* pETCtsR: *O. oeni* CtsR production	*Hin*dIII	This study
ctsR5	CCGGGAGGATCCAAAGGAGGGGGTTGAATG		*Bam*HI	This study
Olcg16	ATCGGCGGATCCTATCAAATACCTCCTATTAACTAA	pDL*hsp18*: *hsp18* promoter region for transcriptional fusion with *bgaB* in *B. subtilis*	*Bam*HI	(Grandvalet et al., 2005)
Olcg20	GCCCGAATTCTAAATTAATCGAAGCCTTTTGAC		*Eco*RI	
Olcg302	GGGCCATGGCAAATACCTCCTGATTCATTAATGCAGGGGTAC	pSIPSYN: Synthetic promoter P*_SY N_* amplification	*Nco*I	(Darsonval et al., 2016)
Olcg303	CCCAAGCTTGCGCAACTGTTGGGAAGGG		*Hin*dIII	

**Over-expression in *E. coli* BL21**				

Olcg1	ATGCATGCCATGGCAGAAGCTAATATTTCAG	pET*ctsR* : *O. oeni* ctsR coding sequence into pET28a(+) for overexpression in *E. coli* BL21	*Nco*I	This study
Olcg2	GGGCTCGAGTCCATGAATGTTTGTACTCTCA		*Xho*I	
hsp18N	GGGCCATGGCAAATGAATTAATGGATAGA	pET*hsp18 : O. oeni hsp18* coding sequence into pET28a(+) for overexpression in *E. co*li BL21	*Nco*I	This study
hsp18S	GGGGAGCTCTTATTGGATTTCAATATGATGAGT		*Sac*I	

**Bacterial Two-Hybrid system in *E. coli***				

CtsR21	GGAGGATCCCGCAGAAGCTAATATTTCAGATTT	*ctsR* coding sequence,T18-*ctsR* and T25-*ctsR* fusions	*Bam*HI	This study
CtsR20	GGTGGTCTCGAATTC**TTA**TCCATGAATGTTTGTACTCTCAA		*Eco*RI/*Bsa*I	
CtsR25	AAGAAGCTTATTGATAGGAGGATCAAATT*ATG*GCAGAAGCTAATA	*ctsR* coding sequence, *ctsR-*T18 and *ctsR-*T25fusions	*Hind*III	This study
CtsR22	GGAGGATCCTCTCCATGAATGTTTGTACTCTCAA		*Bam*HI	
ClpL1-21	GGAGGATCCCGCTAATAATGATTATTTTAATAACAG	*clpL1* coding sequence,T18-*ctsR* and T25-*ctsR* fusions	*Bam*HI	This study
ClpL1-20	CTCGAATTC**TCA**CTTTCCAGTGATCTTAATTG		*Eco*RI	
ClpL1-23	GGTGGTCTCAAGCTTATTGATAGGAGGATTCAATC*ATG*	*clpL1* coding sequence, *clpL1-*T18 and *clpL1-*T25fusions	*Hin*dIII/*Bsa*I	This study
ClpL1-22	GGAGGATCCTCCTTTCCAGTGATCTTAATTGTTC		*Bam*HI	
ClpL2-21	GGTGGTCTCGGATCCCGCTGATTATAATGACGATCCCT	*clpL2* coding sequence,T18- *clpL2* and T25-*clpL2* fusions	*Bam*HI/*Bsa*I	This study
ClpL2-20	GGTGGTCTCGAATTC**TTA**ATTATCTTTTTGATTCGTGGCGG		*Eco*RI/*Bsa*I	
ClpL2-27	GGTGGTCTCAAGCTTAGGAGGAGGAAAAA*ATG*GCTGATTATAATGACGATCCCT	*clpL2* coding sequence, *clpL2-*T18 and *clpL2-*T25 fusions	*Hin*dIII/*Bsa*I	This study
ClpL2-22	GGTGGTCTCGGATCCTCATTATCTTTTTGATTCGTGGCGG		*Bam*HI/*Bsa*I	
ClpC21	GGTGGTCTCCTGCAGGATAATCAATACACAGCATCTG	*clpC* coding sequence, T25-*clpC* fusions	*Pst*I	This study
ClpC20	GGTGGTCTCTCTAGA**TTA**TTTTGTACTTTTTTCAATTTGGTG		*Xba*I	
ClpC21C	GGTGGTCTCCTGCAGCGATAATCAATACACAGCATCTG	*clpC* coding sequence (with clpC20), T18-*clpC* fusions	*PstI*	This study
ClpC21KT	GGTGGTCTCCTGCAGCCGATAATCAATACACAGCATCTG	*clpC* coding sequence (with clpC20), T25-*clpC* fusions	*PstI*	This study
ClpC27	CCCAAGCTTAGGAGGAAACATTC*ATG*GATAATCAATACACAGCATCTG	*clpC* coding sequence, *clpC-*T18 and *clpC-*T25 fusions	*Hin*dIII	This study
ClpC24	GGTGGTCTCCTGCAGTCTTTTGTACTTTTTTCAATTTGGTG		*Pst*I	

*^1^Forward primers are numbered with odd numbers and Reverse primers with even numbers; ^2^Restriction sites are underlined; and ^3^ATG codons are in italic and STOP codon appears in bold.*

### DNA Manipulation and Bacterial Transformation

*Oenococcus oeni* genomic DNA was extracted using the InstaGene^TM^ Matrix (Bio-rad, Hercules, CA, United States), PCR amplifications for cloning constructions were performed with Expand High Fidelity PCR System (Roche, Meylan, France) and colony PCR amplifications were performed with GoTaq^®^DNA polymerase (Promega, Charbonnières-les-bains, France). Plasmids from *E. coli* were prepared using a GeneJET Plasmid Miniprep Kit (Thermo Fisher Scientific, Illkirch, France). PCR DNA fragments were purified with GeneJET PCR Purification Kit (Thermo Fisher Scientific). T4 DNA ligase and restriction endonucleases were purchased from New England Biolabs Inc. (NEB, Evry, France). Plasmids and ligation products were transferred by electroporation into *E. coli* strains (Taketo, 1988). Briefly, *E. coli* cells in early exponential phase (OD_600_ = 0.5) were collected from 500 ml LB culture, washed twice in 250 ml of sterile ice-cold ultra-pure water and concentrated 100-fold in 10% glycerol. Aliquots of 0.1 ml were mixed on ice with plasmid DNA or ligation mixture, and then submitted to an electroporation pulse of 25 μF, 200 Ω, and 12.5 kV/cm. After the pulse, cells were directly mixed with 1 ml fresh LB medium, incubated for 20 to 40 min at 37°C and then plated on agar LB medium supplemented with suitable antibiotic. Plasmids were transferred by electroporation into *O. oeni* as previously described (Assad-García et al., 2008). *O. oeni* recombinant strains were selected on FT80m plates supplemented with erythromycin, vancomycin and lincomycin (20 μg ⋅ ml^−1^ each). *B. subtilis* was transformed with recombinant vectors as previously described by Msadek et al. (1998). Transformants were selected on agar LB medium supplemented with suitable antibiotics.

### Plasmid Constructions and Cloning Strategy

Plasmids and primers used in this study are respectively listed in Tables 1, 2.

The plasmid pSIPSYNASCtsR was constructed by inserting in reverse orientation the amplified full-length coding sequence of the *O. oeni ctsR* gene downstream from the synthetic P_SY N_ promoter of plasmid pSIPSYN. The *ctsR* gene was amplified by PCR from *O. oeni* ATCC BAA-1163 genomic DNA using ASCtsR3 and ASCtsR6 primers (Table 2) generating *Nco*I (3′-end of *ctsR*) and *Sma*I (5′-end of *ctsR*) restriction sites. The resulting plasmid named pSIPSYNASCtsR, carrying the complete coding sequence of the *ctsR* gene in reverse orientation under the control of the synthetic P_SY N_ promoter_,_ was transferred by electroporation into *O. oeni.* The native pSIPSYN vector, without any insert, was introduced into *O. oeni* to serve as a control. The corresponding *O. oeni* recombinant strains were respectively designated by OoASctsR and Oosyn. The presence of the vector was confirmed by colony PCR amplification with specific primers Olcg303, olg302, or ASCtsR3. One colony was suspended in 50 μl of lysis buffer (100 mM EDTA, [pH8], 20 mg ⋅ ml^−1^ Proteinase K) and then mixed by vortex. The resulting mix was heated 15 min at 65°C then 5 min 95°C and 5 μl of this mix were used as a DNA template in the PCR mix.

The pET28a(+) plasmid (Novagen, VWR International S.A.S, Fontenay-sous-Bois, France) was used for *cstR* and *hsp18* overexpression in *E. coli* BL21 λ(DE3) strain. The *ctsR* and *hsp18* coding sequences were amplified by PCR using primer pairs olcg1 and olcg2 and hsp18N and hsp18S, respectively. PCR products were respectively cloned between the *Nco*I and *Xho*I sites and the *Nco*I and *Sac*I sites of pET28a(+). The resulting plasmids, pET*ctsR* and pET*hsp18*, were then introduced into in *E. coli* BL21 λ(DE3) by electroporation.

The pXT integrative plasmid was used to express the *O. oeni cstR* in the *B. subtilis* Δ*ctsR* mutant strain (Derré et al., 2000). The full-length coding sequence was amplified by PCR from *O. oeni* ATCC BAA-1163 genomic DNA using primers CtsR2 and CtsR5 (Table 2). The PCR-generated fragment was cloned between the *Bam*HI and *Hin*dIII sites of plasmid pXT under the control of a xylose inducible promoter (P*_xylA_*). The resulting plasmid, pXTCtsR25, was integrated at the *B. subtilis thrC* locus of the Δ*ctsR*-*hsp18’*-*OoctsR* strain, corresponding to the Δ*ctsR* mutant strain (QB4991) carrying a transcriptional fusion between the promoter region of the *O. oeni hsp18* gene and the *bgaB* gene of *Geobacillus stearothermophilis* (pDL*hsp1*8→QB4991) (Grandvalet et al., 2005). The recombinant strains were first selected for resistance to spectinomycin and screened for susceptibility to erythromycin and colony PCR amplifications were performed with primers CtsR2 and CstR5 to confirm successful chromosomal integration.

To construct recombinant plasmids used in the BACTH complementation assays, genes coding for the *O. oeni* proteins were amplified by PCR using appropriate primers pairs (Table 2) and genomic DNA from *O. oeni* ATCC BAA-1163 as the template. The resulting PCR products were cloned between the *Bam*HI and *Eco*RI sites of the pKT25 and pUT18C vectors (except for the *clpC* gene, cloned between the *Pst*I and *Xba*I sites). The resulting plasmids expressed hybrid proteins, in which the proteins of interest were fused to the C-terminus of the T25 or T18 fragment of adenylate cyclase (AC) from *Bordetella pertussis*. For the reciprocal combinations, PCR-generated fragments (without the stop codon, see Table 2) were digested with appropriate enzymes and cloned between the *Hind*III and *Bam*HI sites of the pKNT25 and pUT18 vectors (except for *clpC* which was cloned between the *Hind*III and *Pst*I sites). This second set of recombinant plasmids expressed hybrid proteins in which the proteins of interest were fused to the N-terminus of the T25 or T18 fragment of AC.

### DNA Sequencing and Sequence Analysis

Nucleotide sequences of recombinant vector inserts were verified on both strands by nucleotide sequencing with labeling and capillary separation on the AB3730xl performed by GeneWiz^®^Europe (Essex, United Kingdom). Nucleotide sequencing results were analyzed using Clustal Omega software^2^.

### Production and Purification of CtsR and CtsR Polyclonal Antibody Production

CtsR was purified from *E. coli* BL21-CtsR strain, as described previously (Assad-García et al., 2008). Briefly, *E. coli* BL21-CtsR was grown aerobically in LB medium supplemented with 50 μg ⋅ ml^−1^ kanamycin at 37°C until OD_600 =_ 0.7–1. Isopropyl-β-D-thiogalactopyranoside (IPTG, 1 mM) was added to the culture to induce recombinant CtsR synthesis. After 15 h aerobic incubation (150 rpm) at 21°C, cells were harvested by centrifugation (6,500 × *g* for 10 min). The pellet was suspended in cold lysis buffer (50 mM Na_2_HPO_4_/NaH_2_PO_4_ [pH 8], 300 mM NaCl) and disrupted in a constant cell disruption system (Cell-D, Constant Systems Ltd., Roquemaure, France) with glass beads (0.5 μm). The suspension was centrifuged at 6,300 × *g* for 20 min at 4°C to remove unbroken cells and cell debris. The supernatant was loaded onto a 1-ml Ni-Nitrilotriacetic acid column (Qiagen, Courtaboeuf, France) equilibrated with lysis buffer. The column was washed 10 times with 1 column volume of lysis buffer supplemented with 20 mmol ⋅ l^−1^ imidazole. The recombinant CtsR protein was then eluted with lysis buffer supplemented with 250 mmol ⋅ l^−1^ imidazole. The eluate was dialyzed against lysis buffer and protein purification was monitored by sodium dodecylsulfate-polyacrylamide gel electrophoresis (SDS–PAGE, 12.5% polyacrylamide) as described by Laemmli (1970). Polyclonal antibodies were obtained by direct immunization of SPF-rabbits with purified CtsR protein (Eurogentec, Liège, Belgium). Antiserum was used for CtsR immunodetection.

### Whole Cell Extracts and Western Blotting

*Oenococcus oeni* Lo18 and CtsR protein levels were detected using rabbit antiserum raised against Lo18 (laboratory stock) or CtsR (obtained as described above). In both cases, the pellet from 50 ml of culture was washed twice in saline (9 g ⋅ l^−1^ NaCl) and adjusted to 30 OD Unit ⋅ ml^−1^ in lysis buffer (10 mM Tris–HCl [pH 8]). Cells were disrupted by two consecutive treatments using a Precellys homogenizer with glass beads (0.5 μm) at 6,500 × *g* (Precellys, Paris, France; 60 s–20 s pause-60 s). The suspension was centrifuged at 13,200 × *g* for 15 min at 4°C to remove unbroken cells and cellular debris. Supernatants containing total cellular proteins were collected and assayed with Bio-rad Protein Assay Dye Reagent Concentrate (Bio-Rad) with bovine serum albumin as the standard. Total cellular proteins (15 μg) were mixed with loading buffer 5X (250 mM Tris–HCl [pH 8], 50% glycerol, 77 g ⋅ l^−1^ DTT, 0.4% bromophenol blue, 10% SDS), heated 5 min at 85°C and separated by SDS-PAGE (17% polyacrylamide). Proteins were then transferred on a nitrocellulose membrane (BioRad, Les Ulis, France) using a Trans-Blot Turbo (BioRad). The membrane was saturated in blocking buffer (1X PBS, 0.2% Tween^®^20 and 5% Bovine Serum Albumin, BSA) and hybridized in blocking buffer with rabbit antiserum, containing polyclonal antibodies directed against *O. oeni* Lo18 (1:1,000) or CtsR (1:1,000), for one night at room temperature with gentle shaking. The membrane was then washed three times for 5 min in 1X PBS-T (0.2% Tween^®^20) and incubated one hour at room temperature with conjugated fluorophore IRDye 680LT goat anti-rabbit antibodies (1:10,000, LiCor^®^, Biosciences-GmbH) in blocking buffer. The membrane was washed again in 1X PBS-T at room temperature three times for 5 min. Detection was performed with Odyssey Fc Western Blot Imaging System (LiCor^®^Biosciences-GmbH).

### β-Galactosidase Assays in *B. subtilis*

Overnight cultures of *B. subtilis* grown in LB medium supplemented with chloramphenicol (5 μg ⋅ ml^−1^) were diluted to OD_600_ = 0.05 in fresh LB medium and grown at different temperatures 28, 37, or 42°C under aerobic conditions (140 rpm). At the mid-exponential phase (OD_600_ = 1.5), xylose was added at a final concentration of 20 mM to induce *ctsR* gene expression during 12 h (DO_600_ = 3). For each sample, β-galactosidase activity was determined as previously described (Miller, 1972; Grandvalet et al., 2005) and expressed as Miller units per mg cellular protein. Protein concentrations were determined using the Bio-Rad protein assay. Experiments were performed three times on three independent cultures.

Repression of *hsp18’-bgaB* expression by CtsR was tested at six different temperatures by following β-galactosidase activity LB agar plates supplemented with 5-bromo-4-chloro-3-indolyl-β-D-galactopyranoside (X-Gal), with 20 mM xylose to induce expression of the *O. oeni ctsR* gene. Strains were plated in parallel with incubation at six different temperatures: 28, 33, 35, 37, 42, and 50°C.

### BACTH Assays

For BACTH assays, recombinant vectors (pKT25, pKNT25, pUT18C, and pUT18) carrying the studied *O. oeni* genes were co-transformed in all possible combinations into *E. coli* DHT1 cells (Dautin et al., 2000). Co-Transformants were plated on LB agar medium supplemented with 40 μg ⋅ ml^−1^ X-Gal and 0.5 mM IPTG (isopropylthio-β-galactoside) and incubated at 28°C for 24 to 36 h. Interaction efficiencies between different hybrid proteins were quantified by measuring β-galactosidase activity in a 96-well microtiter plate after cell permeabilization. For each co-transformation combination, six independent clones were tested. *E. coli* co-transformed clones were grown in 300 μl LB broth supplemented with 0.5 mM IPTG, 50 μg ⋅ ml^−1^ kanamycin and 100 μg ⋅ ml^−1^ ampicillin in 5 ml hemolysis tube and then incubated at 28°C for 16 to 24h under aerobic conditions (150 rpm). Cultures were diluted 1:5 into M63 medium (Sambrook et al., 1989) into a final volume of 200 μL and OD_595_ was measured by GENios reader (Tecan, Lyon, France). Cell suspensions were replaced by LB medium diluted 1:5 into M63 medium for control wells. Cells were permeabilized by adding 7 μl SDS (0.05%, *w*/*v*) and 10% chloroform per well with vigorous mixing with a multichannel pipette and incubated 30 to 40 min at room temperature. For enzymatic assays, 20 μl of permeabilized cells were added to 105 μl of reaction mixture: 70 mM Na_2_HPO_4_, 30 mM NaH_2_PO_4_, 1 mM MgSO_4_, 0,2 mM MnSO_4_ [pH7.0], 100 mM β-mercaptoethanol and 0.1% ONPG (o-nitrophenyl-β-D-galactopyranoside). After 30 min at room temperature, OD_405_ was measured using a GENios reader (Tecan). Enzymatic activities, *A*, were calculated in relative units using the following formula:

$$A_{r.u}=1,000\times \frac{\left({\mathrm{OD}}_{405}-{\mathrm{OD}}_{405_{\mathrm{control well}}}\right)}{\left({\mathrm{OD}}_{595}-{\mathrm{OD}}_{595_{\mathrm{control well}}}\right)}/{\mathrm{Incubation time}}_{\min}$$

### Statistical Analysis

The significance of the difference among percentage of cultivability values was determined by a two-tailed Student *t*-test. The confidence interval for a difference in the means was set at 95% (*P* ≤ 0.05) for all comparisons.

## Results

### Antisense RNA Approach in *O. oeni* to Characterize CtsR Function *in vivo*

**FIGURE 1:**
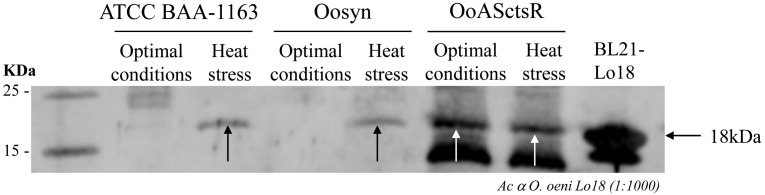
Immunodetection of Lo18 in *O. oeni* wild type strain (ATCC BAA-1163) and recombinant strains carrying the control pSIPSYN plasmid (Oosyn) or pSIPSYNASctsR plasmid allowing expression of asRNA targeting *ctsR* (OoASctsR) were cultivated at 28°C in FT80m medium up to the end of the exponential growth phase. Total cellular proteins were extracted immediately (28°C) or following a sub-lethal thermal treatment (30 min at 42°C). For each strain and each condition, 15 μg of protein were separated by SDS-PAGE. The *O. oeni* Lo18 protein was detected by immunodetection with polyclonal antibodies directed against Lo18 and anti-rabbit antibodies conjugated with IRDye 680LT fluorophore (LiCor^®^) as secondary antibody. Total protein extract from *E. coli* BL21λDE3 carrying pET*hsp18* plasmid was used as a positive control (BL21-Lo18).

The antisense RNA approach is the only method currently available to modulate gene expression in *O. oeni* (Darsonval et al., 2016). Investigation of *ctsR* gene function in *O. oeni* was therefore performed by producing antisense RNA (asRNA) targeting *ctsR* mRNA. *O. oeni* was transformed with the recombinant plasmid encoding asRNA targeting the full-length *ctsR* mRNA (pSIPSYNASctsR), and the empty control plasmid (pSIPSYN), giving strains OoASctsR and Oosyn, respectively. In order to validate the efficiency of this asRNA approach, the effect of ASctsR RNA production was examined at the protein level by immunodetection of Lo18 (Figure 1). Lo18 is a well-studied small Hsp encoded by *hsp18*, chosen as a representative of CtsR regulon expression. Under optimal growth-conditions, Lo18 is not detected in the wild type strain ATCC BAA-1163 and the recombinant strain Oosyn, carrying the control plasmid, due to repression by CtsR. Consistent with this observation, Lo18 is detected after heat stress in both strains, indicating that the CtsR-dependant stress response regulation is functional in these conditions (Figure 1). In OoASctsR, Lo18 protein is detected in both conditions, with and without thermal stress. This observation indicates derepression of CtsR-controlled genes in the absence of heat stress due to the production of ASctsR RNA. Protein bands with an apparent molecular mass smaller than Lo18 are detected in both OoASctsR and *E. coli* overexpressing the *hsp18* gene. These correspond to truncated forms of Lo18 as previously reported by Coucheney et al. (2005a).

**FIGURE 2:**
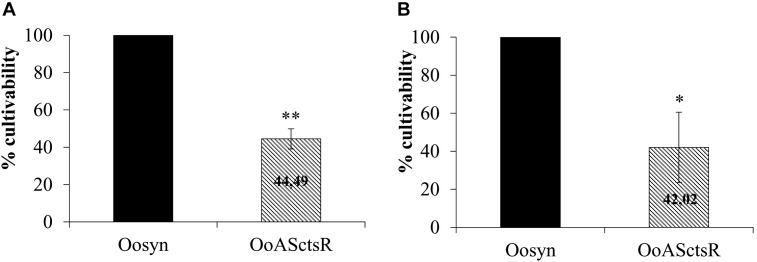
Cell cultivability following stress treatment. Recombinant strains carrying the control empty vector (Oosyn; black bars) or plasmid with asRNA targeting *ctsR* (OoASctsR; hatched bars) were grown at 28°C until mid-exponential phase. Cultures were transferred to 48°C **(A)** or into acidified FT80m medium (pH 3.5) **(B)**. Cultivability was estimated on agar plates (CFU ⋅ ml^−1^) after 90 min treatment. After CFU counting on agar plates, the cultivability rate is calculated by dividing the number of cells following treatment by total number of cells before stress treatment, normalized to the reference strain Oosyn set at 100%. Significant differences are based on a unilateral and paired *t*-test (^∗∗^*P* < 0.005; ^∗^*P* < 0.05).

Since this antisense RNA approach leads to derepression of the CtsR regulon, we tested survival of the bacteria following stress treatment (Figure 2). No loss of cultivability was observed for the Oosyn control strain 90 min after temperature shifting from 28 to 48°C (Figure 2A) or a shift in pH from 5.3 to 3.5 (Figure 2B). In contrast, 56% of the OoASCtsR cells are lost following heat stress (Figure 2A) and 58 % after pH 3.5 acid stress (Figure 2B). This result suggests that derepression of the CtsR regulon in the absence of stress interferes with cell survival under stress conditions, indicating that CtsR plays a key role in the *O. oeni* stress response.

### CtsR of *O. oeni* Represses *hsp18* Expression

*Bacillus subtilis* was used as a tool for the functional analysis of *O. oeni* CtsR. The *B. subtilis* QB4991 Δ*ctsR* mutant strain (Derré et al., 1999) was used to express the *O. oeni ctsR* gene in single copy from a xylose-inducible promoter (strain QB4991-XT*OoctsR*; see Materials and Methods). In order to assess the functionality of *O. oeni* CtsR in *B. subtilis*, a transcriptional fusion between the promoter region of *O. oeni hsp18* and the *bgaB* thermostable β-galactosidase gene (*hsp18’-bgaB*) was integrated in single copy in the *B. subtilis* wild type and QB4991-XT*OoctsR* strains, respectively designated WT and Δ*ctsR-*XT*OoctsR*.

**FIGURE 3:**
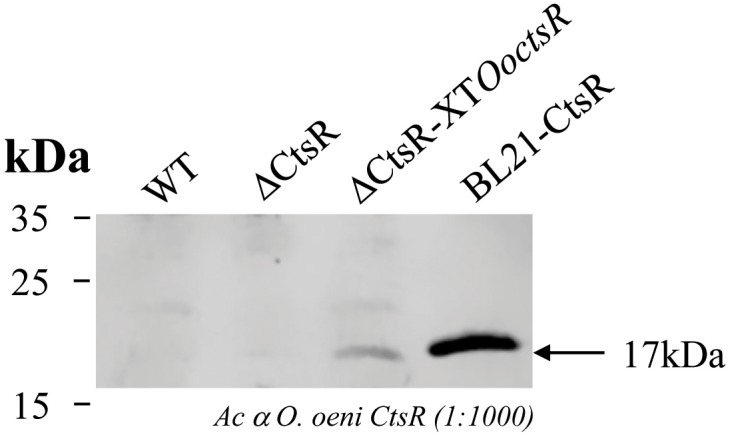
Immunodetection of the *O. oeni* CtsR protein in *B. subtilis*. *B. subtilis* wild-type (WT), *ctsR-*deficient (Δ*ctsR*) and *ctsR*-deficient expressing the *O. oeni ctsR* gene (Δ*ctsR-*XT*OoctsR*) strains were grown at 28°C in LB medium and xylose (20 mM) was added at the mid-exponential growth phase (OD_600_ = 1.5). Cells were harvested 12 h after adding xylose and total cellular proteins were extracted and analyzed by Western blot using polyclonal antibodies directed against CtsR from *O. oeni* and anti-rabbit antibodies conjugated with IRDye 680LT fluorophore (LiCor^®^) as secondary antibody. Total protein extract from *E. coli* BL21 carrying pET*ctsR* plasmid (BL21-CtsR) strain was used as a positive control.

Expression of the *O. oeni* CtsR protein in *B. subtilis* was verified by Western blot (Figure 3). *B. subtilis* wild-type (WT), Δ*ctsR* mutant (Δ*ctsR*) and Δ*ctsR* mutant with the xylose-inducible *O. oeni ctsR* gene (Δ*ctsR-*XT*OoctsR*) strains were grown at 28°C in LB with xylose added in the mid-log phase. No signal was detected in whole cell extracts from the wild type or Δ*ctsR* mutant strains whereas a band with a 17 kDa apparent molecular mass, corresponding to that of the CtsR positive control (BL21*-*CtsR), was detected in the strain carrying the *O. oeni ctsR* gene under the control of P*xylA* promoter, confirming correct heterologous expression.

Expression of the *hsp18’-bgaB* fusion in *B. subtilis* strain Δ*ctsR-*XT*OoctsR* was followed by β-galactosidase activity assays during growth at 28°C in LB broth medium with or without xylose. In the absence of xylose, the *hsp18*’-*bgaB* fusion is strongly expressed (approximately 350,000 Miller units ⋅ mg^−1^). In the presence of xylose, expression of the *hsp18’*-*bgaB* fusion is repressed more than 10-fold (34,000 Miller units ⋅ mg^−1^). Taken together, these results indicate that the *O. oeni* CtsR repressor is fully functional in *B. subtilis* and acts as a repressor of *hsp18* expression.

**Table 3:** BACTH analysis of CtsR, ClpL1, ClpL2, and ClpC interactions^1^.

	Units of β-galactosidase activity							
Hybrid protein	CtsR-T25	T25-CtsR	ClpL1-T25	T25-ClpL1	ClpL2-T25	T25-ClpL2	ClpC-T25	T25-ClpC
CtsR-T18	**271**	**236**	37	24	33	11	24	25
T18-CtsR	**203**	**458**	**88**	**92**	9	26	38	35
ClpL1-T18	**72**	**62**	**192**	**120**	**196**	11	14	31
T18-ClpL1	26	24	**70**	**116**	17	**111**	10	18
ClpL2-T18	18	12	18	30	21	**54**	14	20
T18-ClpL2	14	17	13	30	17	25	11	30
ClpC-T18	34	15	13	14	19	7	**140**	17
T18-ClpC	22	13	16	32	10	8	49	**64**

*^1^Functional interaction between the indicated hybrid proteins was quantified by measuring β-galactosidase activities in E. coli DHT1 cells harboring the corresponding plasmids, as described in Materials and Methods and listed in Table 1. Hybrid proteins in which the O. oeni proteins are fused to T25 (or T18) via their N-terminal extremity are marked as T25-X (or T18-X). Hybrid proteins in which the O. oeni proteins are fused to T25 (or T18) via their C-terminal extremity are marked as X-T25 (X-T18). Each result represents the mean value of six independent measurements. Under the same assay conditions, functional complementation between T25-Zip and T18-Zip, used as a positive control yielded about 400 units of β-galactosidase (Karimova et al., 1998). Under the same conditions, cells expressing non-fused fragments (T25 and T18 associations), used as negative control gave about 20 units of β-galactosidase. β-galactosidase activity levels at least 2- to 3-fold higher than those measured in DHT1 negative control cells are considered as effective protein–protein interactions and appear in bold and highlighted in gray.*

### CtsR of *Oenococcus oeni* Acts as a Thermosensor

**FIGURE 4:**
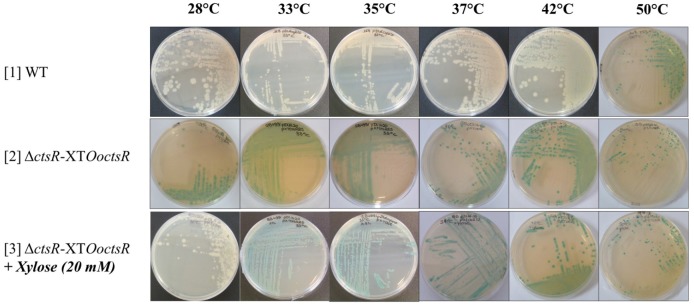
The *O. oeni* CtsR repressor is a specific thermosensor *in vivo*. The *O. oeni ctsR* gene was expressed in the *B. subtilis* Δ*ctsR* mutant strain (Δ*ctsR-*XT*OoctsR*) under the control of a xylose-inducible promoter. Repression of *hsp18’-bgaB* expression by CtsR was monitored at different temperatures (28, 33, 35, 37, 42, and 50°C) by blue/white screening on X-Gal plates.

As previously suggested by Derré et al. (2000) and confirmed in *L. lactis, G. stearothermophilus*, and *B. subtilis* by Elsholz et al. (2010), CtsR is an intrinsic heat sensor with a species-specific temperature threshold. We tested thermoinduction of *hsp18* by *O. oeni* CtsR using *B. subtilis* as a heterologous host. Expression of *hsp18*’-*bgaB* was followed by measuring β-galactosidase activities of the wild type (WT) and Δ*ctsR-*XT*OoctsR B. subtilis* strains during growth at different temperatures (28, 33, 35, 37, 42, and 50°C) with blue/white screening on X-Gal containing LB-agar plates with or without added xylose (Figure 4). White colonies correspond to the transcriptional repression of the *hsp18’-bgaB* fusion by CtsR. Blue colonies indicate thermoinduction of *hsp18’-bgaB* expression following CtsR inactivation. We have previously shown that CtsR of *B. subtilis* is able to repress expression of *O*. *oeni hsp18* (Grandvalet et al., 2005). As expected, in wild-type *B. subtilis* strain (Figure 4, lane [1], WT), the CtsR regulator is active and represses *hsp18’-bgaB* expression during growth at temperatures ranging from 28 to 42°C. Following incubation at 50°C, expression of *hsp18’-bgaB* is induced due to inactivation of CtsR. As previously reported, these results confirm that the *B. subtilis* CtsR is active at temperatures up to 42°C (Elsholz et al., 2010). In the strain expressing *O*. *oeni* CtsR (Δ*ctsR-*XT*OoctsR*), *hsp18’-bgaB* is expressed at all growth temperatures in the absence of xylose, (Figure 4, lane [2]) due to absence of CtsR repression. When the *O. oeni ctsR* gene is expressed by adding xylose (Figure 4, lane [3] Δ*ctsR-*XT*OoctsR*+ Xylose) the *hsp18*’-*bgaB* fusion is repressed during growth at 28°C. However, repression by *O. oeni* CtsR no longer occurs during growth at all temperatures tested above 28°C (33, 35, 37, 42, and 50°C) indicating that *O. oeni* CtsR is active at 28°C, the optimal growth temperature for *O. oeni*, and totally inactive at 33°C and above. These results strongly indicate that the *O. oeni* CtsR transcriptional repressor is an intrinsic heat sensor with a specific temperature threshold adapted to the natural habitat of *O. oeni*.

### BACTH Analysis of CtsR/ClpL1/ClpL2/ClpC Interactions

In order to understand the potential protein interaction network involved in regulation of CtsR activity in *O. oeni*, we used the BACTH technique. This approach is based on the interaction-mediated reconstruction of a cyclic AMP (cAMP) signaling pathway in *E. coli*. Candidate proteins are produced as translational fusions with two fragments (T25 and T18) of the catalytic domain of *Bordetella pertussis* Adenylate Cyclase (AC) in an *E. coli* DHT1 *cya^−^* AC-deficient strain (Karimova et al., 1998). Upon interaction of the hybrid proteins, AC activity is restored due to the spatial proximity of the T25 and T18 fragments, leading to cAMP synthesis and transcriptional activation of catabolic operons such as the well-known lactose operon.

To characterize physical interactions, the four proteins (CtsR, ClpL1, ClpL2, and ClpC) were methodically tested for pairwise interactions using the BACTH complementation assay. The DNA sequences encoding the proteins of interest were cloned into appropriate BACTH vectors to generate hybrid proteins fused either at the N-terminal (pKT25 or pUT18C) or C-terminal extremities (pKNT25 or pUT18) of either the T25 or the T18 fragment of the *B. pertussis* AC (see Materials and Methods). To analyze putative physical associations between the resulting hybrid proteins, the *E. coli* DHT1 *cya^−^* strain was cotransformed with pairs of recombinant plasmids (Table 1) expressing the T25 and the T18 hybrid proteins (pKT25/pUT18C, pKT25/pUT18, pKNT25/pUT18C, or pKNT25/pUT18). The efficiency of functional complementation between the different hybrid proteins was determined by β-galactosidase assays and the results of the different pairwise combinations are summarized in Table 3.

Based on the characteristic features of CtsR and ClpATPases in the *Firmicute* phylum, we would anticipate homodimerization of CtsR and the oligomerization of the three ClpATPases. As expected, self-interaction of CtsR is seen with the four combinations of the hybrid proteins with β-galactosidase activities ranging from 203 to 458 units (Table 3). The BACTH analysis data also confirmed self-association of the ClpL1 and ClpC ATPases, but for ClpL2 self-association was less obvious. Among the four pairwise combinations of the hybrid proteins, a single one led to β-galactosidase activity indicating a possible self-association, but reaching only 54 units. These observations suggest either weak physical interactions or that ClpL2 may require additional partners to form proper oligomers.

The BACTH results suggest that ClpC is likely to form symmetric homo-oligomers. Indeed, the ClpC-T25 hybrid protein, in which ClpC is C-terminally fused with T25, interacts efficiently with the ClpC-T18 hybrid protein, with a free ClpC N-terminus (140 units) but not with T18-ClpC (49 units).

Our analysis also revealed that the CtsR repressor associates with ClpL1. For CtsR and ClpL1, four out of eight possible pairwise combinations of the hybrid proteins led to β-galactosidase activities ranging from 62 to 92 units, indicating efficient physical interaction between these two proteins.

The BACTH analysis also highlights that ClpL1 is also able to interact with ClpL2. As shown in Table 3, T25-ClpL2 associates with T18-ClpL1, leading to β-galactosidase levels 10 times higher than with ClpL1-T18 (111 and 11 units, respectively) suggesting a possible symmetric hetero-oligomer formation. Taken together, these results suggest that ClpL1 may play a crucial role in regulation of CtsR activity during stress response in *O. oeni*.

## Discussion

Due to poor transformability and the lack of efficient genetic tools, *O. oeni* is not readily genetically tractable. Directed mutagenesis in *O. oeni* remains impossible to date and little is known about its genetic regulatory mechanisms. The asRNA production approach is presently the only approach to investigate stress response in this bacterium (Darsonval et al., 2016). By targeting *ctsR* for knockdown, we undertook an *in vivo* approach with the aim of broadening our knowledge on *O. oeni* CtsR, the only stress response regulator described to date. In this work, *ctsR* inhibition by asRNA affects expression of *ctsR* regulon genes leading to derepression under optimal growth conditions, as shown by an increased Lo18 protein levels at 28°C (Figure 1). This confirms for the first time *in vivo* CtsR-dependent regulation of *hsp18* gene expression in *O. oeni*. We also highlighted that expression of a complementary asRNA targeting the full-length of *ctsR* mRNA reduced cultivability of *O. oeni* by 50 % following sub-lethal temperature or acid stress exposure (Figure 2). Our data indicate that CtsR plays an essential role in the stress response process with a crucial involvement in coping with prolonged heat and acid stress. Regulation by CtsR may extend beyond a specific role in thermoprotection or acid-tolerance since its inactivation in *Lactobacillus plantarum* leads to pleiotropic effects correlated with its obvious housekeeping function as a central stress response regulator in *O. oeni* (van Bokhorst-van de Veen et al., 2013). We show here that *ctsR* inhibition conferred a heat- and acid-sensitive phenotype to the recombinant strain. Interestingly, we previously observed this phenomenon by inhibiting *hsp18* expression with the same RNA silencing approach (Darsonval et al., 2016). These results suggest that in LAB, stress response gene expression must be carefully fine-tuned: levels that are too low under stress conditions or too high under optimal conditions would be deleterious. Indeed, whereas in most Gram-positive bacteria described so far, *ctsR* deletions lead to an increase in heat resistance and general stress tolerance (Nair et al., 2000; Chastanet et al., 2001; Karatzas and Bennik, 2002; Hüfner et al., 2007; Zotta et al., 2009), in *L. plantarum*, incubation at 40°C strongly inhibited the growth of the mutant strain without affecting the wild type (Fiocco et al., 2010). These unexpected acid- and heat-sensitive phenotypes observed in *O. oeni* OoASCtsR (Figure 2), are in agreement with the observation in *L. plantarum*, where inactivation of *ctsR* causes derepression of *hsp* genes including proteases and molecular chaperones. Excessive levels of Clp proteases and Hsp proteins would be detrimental to the cell, similar to the activity of the acyldepsipeptide antibiotic ADEP4 which has been shown to activate the ClpP protease, resulting in death of growing cells (Brötz-Oesterhelt et al., 2005). For example, accumulation of Lo18, known to be addressed to the membrane under environmental stress conditions, would provoke deleterious effects on physical state of the bacterial cell leading to a stress sensitive phenotype (Fiocco et al., 2010). Indeed, the heat-sensitive phenotype reported for the *L. plantarum*Δ*ctsR* mutant by Fiocco et al led to an intriguing impairment in the cell envelope (Fiocco et al., 2010). Taken together these results raise the possibility that CtsR might control other activities including cell envelope integrity. The partial impact on the cultivability observed suggests the involvement of other regulators of stress response that have not yet been described. In addition, modifications of *ctsR* expression seem to have pleiotropic effects as observed in *L. plantarum* (Fiocco et al., 2010). Further studies will be needed to shed light on this aspect. In *O. oeni*, the combination of Lo18 immune-localization by electron microscopy observation and fluorescence anisotropy could be considered to investigate the impact of CtsR regulon dysregulation on the cell wall and membrane integrity (Coucheney et al., 2005a; Darsonval et al., 2016). Our findings confirm that gene knockdown by antisense RNA is a powerful strategy to study the role of *O. oeni* genes *in vivo*. Obviously, even if this antisense RNA approach is currently the only available technique to modify gene expression in such a genetically intractable organism such as *O. oeni*, it is not a perfect solution.

We began studying the regulation of CtsR activity using *B. subtilis* and *E. coli* as heterologous hosts. In *B. subtilis*, thermo-induction of a *hsp18*’-*bgaB* transcriptional fusion at different temperatures allowed us to show that CtsR can be inactivated in a temperature-dependent manner. These results are in agreement with Derré et al., 1999) and with results obtained in *L. lactis, G. stearothermophilus*, and *B. subtilis* by Elsholz et al. (2010) showing that CtsR is an *in vivo* intrinsic heat sensor with specific temperature thresholds according to the bacterial species. Indeed, CtsR-dependent gene expression is induced at temperatures above 42°C in *L. lactis* but repressed at temperatures up to 50°C in *G. stearothermophilus* (Elsholz et al., 2010). We show here that *O. oeni* CtsR-dependent gene expression is induced at growth temperatures of 33°C and above, acting as a molecular thermometer. CtsR sequences are highly conserved across the phylogenetic group of low-GC% Gram-positive bacteria. Two regions, the HTH and the winged HTH domains, are both conserved and crucial for CtsR activity, Derré et al., 2000) described two point mutants for *B. subtilis* CtsR (V16M and G65S), suppressing CtsR inactivation during heat stress, while Elsholz substituted the Glycine residue 64 by a proline at the tip of the hairpin in the highly conserved glycine-rich loop of the CtsR winged HTH domain and showed that this residue is essential for *B. subtilis* CtsR activity *in vivo* and responsible for CtsR thermosensor ability in several low-GC% Gram-positive bacteria (Derré et al., 2000; Fuhrmann et al., 2009; Elsholz et al., 2010). A piezotolerant strain of *Listeria monocytogenes* resistant to heat, acid and oxidative treatments had a single codon deletion in this conserved glycine-rich hairpin (Karatzas and Bennik, 2002; Karatzas et al., 2003). Nevertheless, while this glycine-rich domain appears to be responsible for CtsR thermosensitivity, it does not seem to be responsible for species-dependent temperature thresholds. Indeed, the glycine-rich domain is highly conserved across *Firmicutes* (Derré et al., 1999). Differences in temperature thresholds may be due to the Hsp machinery specific to each species rather than the glycine-rich region. Regulatory mechanisms of stress response genes have been well investigated in low-GC Gram-positive bacteria and revealed a diversity of actors involved in regulation of CtsR activity (Derré et al., 2000; Nair et al., 2000; Chastanet et al., 2003; Varmanen et al., 2003; Elsholz et al., 2010; Fiocco et al., 2010; Tao et al., 2012; Tao and Biswas, 2013). Namely, *B. subtilis* CtsR is addressed to ClpCP by McsAB complex for degradation above 50°C, whereas in *L. lactis*, ClpE replaces the McsB adaptator and addresses CtsR for degradation above 37°C while the *S. mutans* CtsR is folded and stabilized by ClpL even at room temperature (Derré et al., 2000; Varmanen et al., 2003; Elsholz et al., 2010; Tao and Biswas, 2013). In *O. oeni*, the absence of ClpE, McsA and McsB orthologs may in part explain the difference in CtsR threshold temperature.

Our BACTH analysis indicates an interaction between ClpL1 and CtsR suggesting for the first time in the LAB the involvement of a ClpL family member in regulation of CtsR activity. These findings are consistent with the work of Tao and Biswas in *S. mutans* showing ClpL–CtsR interaction *via* the ClpL D2-small domain at the amino-terminal extremity, a domain conserved in the *O. oeni* ClpL1 sequence (Tao and Biswas, 2013). However, our BACTH analysis did not reveal an interaction between CtsR and ClpL2, which also carries a D2-small region. We noticed that ClpL2 could not form strong homo-oligomers but can interact strongly with ClpL1, possibly by forming hetero-oligomers. ClpATPase proteins usually form hexameric rings of homo- or hetero-oligomers (Ogura and Wilkinson, 2001). This could suggest that ClpL2 might require another partner such as ClpL1 form stable oligomers in our BACTH model and by extension in *O. oeni*. In *L. lactis* and *B. subtilis*, ClpE and McsA present CtsR to a proteolytic complex by interacting with CtsR through their zinc finger motifs, which is absent in ClpL1 (Varmanen et al., 2003; Fuhrmann et al., 2009). One explanation for our observations might be that CtsR and ClpL1 in *O. oeni* interact *via* another domain than D2-small or a Zinc-finger motif. A possible role of ClpX, which has a zinc-finger pattern at its amino-terminal region, cannot be excluded.

The carboxy-terminal region of CtsR may be involved in physical interaction with the chaperone since three out of the four possible BACTH combinations with a free CtsR C-terminal showed interactions with ClpL1. This is the first study to suggest a role for C-terminal domain of CtsR. Indeed, while the amino-terminal region including the dimerization domain, the DNA binding-domain and the thermo-sensing domain, is well-characterized, the role of the C-terminal region is still unknown. It was suggested by Derré et al. that without the C-terminal region, CtsR is sensitive to protease activity and is unstable *in vivo* (Derré et al., 2000). In *O. oeni*, the C-terminal domain of CtsR would be recognized by Clp ATPases to be stabilized, as shown for ClpL in *S. mutans* (Tao and Biswas, 2013).

**FIGURE 5:**
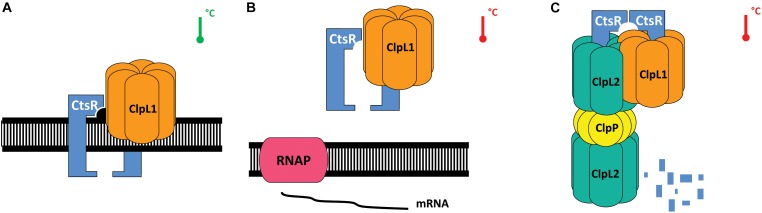
Model for heat-dependent regulation of CtsR activity in *O. oeni* (adapted from Frees et al., 2007; Elsholz et al., 2010) During ideal conditions (depicted by the green thermometer), CtsR is most likely active as a dimer DNA-binding complex and binds to its operator sequences within the promoters of regulon members. ClpL1 binds CtsR to stabilize the regulator on its operator sequence **(A)**. Upon heat exposure (temperature threshold 33°C), represented by the red thermometer, CtsR undergoes a temperature-induced alteration of the winged HTH region that leads to dissociation of CtsR from the DNA **(B)**. Consequently, transcription of the repressed genes is induced. Free CtsR is presumably addressed by ClpL1 **(C)** to the ClpL2P proteolytic complex for degradation, preventing CtsR aggregation. CtsR is then re-activated to repress transcription.

We propose the following model as a model of CtsR activity regulation in *O. oeni* (Figure 5). In agreement with the results of Tao and Biswas showing that ClpL stabilizes CtsR bound to its operator sequence in *S. mutans*, ClpL1 would act similarly under optimal conditions (Figure 5A). Under heat stress conditions, above 33°C, the CtsR–ClpL1 complex would dissociate from the operator site, allowing stress gene expression (Figure 5B). ClpL1 would address inactive CtsR to a proteolytic complex for degradation, such as ClpL2ClpP by interacting with ClpL2 or ClpCP by interacting with ClpC. The involvement of ClpCP in CtsR degradation was previously proposed in a CtsR regulation model (Derré et al., 2000; Elsholz et al., 2010).

Taken together, our results confirmed *in vivo* the central involvement of CtsR in stress response in *O. oeni*, extending an earlier laboratory study using *B. subtilis* as a heterologous host (Grandvalet et al., 2005). Antisense inactivation of *ctsR* expression impacted stress survival of *O. oeni*, confirming CtsR as a master coordinator of general stress response. In addition, we showed that CtsR-dependent gene expression fully induced at 33°C by *O. oeni* CstR identifying CtsR as an intrinsic heat sensor. Furthermore, interaction of CtsR with ClpL1 suggests it is a likely player involved in controlling CtsR activity.

## Author Contributions

MD, FJ, CG, and TM designed the study. MD, FJ, and CG performed the experiments. MD performed the statistical analysis. MD, CG, and TM drafted the manuscript. FJ and HA contributed to the interpretation of the results and writing of the manuscript. All authors contributed to manuscript revision, read, and approved the submitted version.

## Conflict of Interest Statement

The authors declare that the research was conducted in the absence of any commercial or financial relationships that could be construed as a potential conflict of interest.

## Footnotes

1 http://www.ncbi.nlm.nih.gov/nuccore/AAUV00000000.1

2 https://www.ebi.ac.uk/Tools/msa/clustalo/
